# PIKE-R2P: Protein–protein interaction network-based knowledge embedding with graph neural network for single-cell RNA to protein prediction

**DOI:** 10.1186/s12859-021-04022-w

**Published:** 2021-06-02

**Authors:** Xinnan Dai, Fan Xu, Shike Wang, Piyushkumar A. Mundra, Jie Zheng

**Affiliations:** 1 School of Information Science and Technology, ShanghaiTech University, 393 Middle Huaxia Road, Pudong District, Shanghai, 201210 China; 2 Molecular Oncology Group, Cancer Research UK Manchester Institute, The University of Manchester, Alderley Park, Manchester, UK

**Keywords:** Single-cell, Protein prediction, Graph neural network, Knowledge embedding

## Abstract

**Background:**

Recent advances in simultaneous measurement of RNA and protein abundances at single-cell level provide a unique opportunity to predict protein abundance from scRNA-seq data using machine learning models. However, existing machine learning methods have not considered relationship among the proteins sufficiently.

**Results:**

We formulate this task in a multi-label prediction framework where multiple proteins are linked to each other at the single-cell level. Then, we propose a novel method for single-cell RNA to protein prediction named PIKE-R2P, which incorporates protein–protein interactions (PPI) and prior knowledge embedding into a graph neural network. Compared with existing methods, PIKE-R2P could significantly improve prediction performance in terms of smaller errors and higher correlations with the gold standard measurements.

**Conclusion:**

The superior performance of PIKE-R2P indicates that adding the prior knowledge of PPI to graph neural networks can be a powerful strategy for cross-modality prediction of protein abundances at the single-cell level.

## Background

The state of a cell can be described from different perspectives by using a variety of omics data, such as genomic, transcriptomic, and proteomic data [1]. Simultaneous measurement of RNA and protein abundances in the same cells is conducive to the elucidation of cell states [2, 3]. Moreover, there is a correlation between the abundances of RNAs and proteins [4]. According to [5], to some extent, RNAs can guide the expression of proteins. Recently, machine learning methods have been proposed to predict protein abundances from transcriptomic data at the single-cell level. Because the same set of RNAs are used to predict multiple proteins, the task can be formulated in a multi-label machine learning framework. These multi-label models reduce some cost of computation by extracting the general features from input data [6, 7].

Multi-label modeling, which uses one model to predict multiple labels at the same time, has been widely used in machine learning applications, such as image recognition [8] and text classification [9, 10]. Moreover, the multi-label models have been adopted for the prediction of the biological quantities such as the abundances of proteins and RNAs. For example, Liang et al. [11] uses the Gaussian method to identify disease-associated candidate miRNAs; Chou [12] proposes a feature merging method to improve the multiple protein prediction by genomic data; Zou et al. [13] employs a hierarchical neural network for enzyme function prediction. In recent years, graph neural network (GNN) has been one of the most popular core frameworks of the multi-label models [14].

Graph neural networks have been widely applied to different fields, such as natural language processing [15, 16], computer vision [17, 18], and drug discovery [19, 20]. Knowledge graph is a particular application of GNN which introduces knowledge-based information into predictions, boosting performance of GNN on various tasks, such as image classification [21, 22], recommendation systems [23], and dialogue systems [24].

Protein abundance is closely related to other types of molecules in cells, especially RNAs [25–27]. A variety of data sources have been used to predict protein abundance [28, 29]. With the published CITE-seq dataset, machine learning methods have been used to predict protein abundances from RNA expression levels, e.g. [6] proposed a toolkit to study the correlation between the abundances of RNAs and proteins.

Machine learning methods for RNA to protein abundance prediction based on CITE-seq dataset include cTP-net [7] and Random Forest [30]. Zhou et al. proposed cTP-net, using transfer learning to construct a multi-branch model, which predicts the abundances of multiple proteins using the same parameter values [7]. After extracting RNA features, Xu et al. applied the Random Forest models with different parameters for each protein [30] . They found that the Random Forest model achieved higher prediction performance than neural network methods (including cTP-net) on small datasets.

In this work, we propose a novel method called PIKE-R2P (Protein–protein Interaction network-based Knowledge Embedding with graph neural network for single-cell RNA to Protein prediction). Given a sample of scRNA-seq data, the model predicts the abundances of multiple proteins. Our model mainly comprises two parts: a PPI-based GNN and prior knowledge embedding. We use the GNN to capture the relationships among target proteins in sharing some mechanisms of gene expression regulation from transcription to translation. Besides, we integrate the prior knowledge from the STRING database [31] with the model to constrain the protein correlations. PIKE-R2P performs better than existing methods for the protein abundance prediction, especially in terms of accuracy.

## Results

### Dataset

To demonstrate the efficacy of the proposed PIKE-R2P model, we applied it on two CITE-seq datasets available from NCBI GEO database (GSE100866) [4]. The first dataset includes single-cell gene expression of 36,280 mRNAs in 8617 cord blood mononuclear cells (CBMC) with simultaneous measurement of 13 surface proteins. The second dataset contains the expression levels of 29,929 mRNAs and 10 proteins in 7985 peripheral blood mononuclear cells (PBMC).

As these datasets are inherently noisy, we did quality control and noise reduction for them. First, we filtered out cells whose mitochondrial read rates are at least 20%. Then, cells with at most 250 genes expressed were deleted, following the guide of Seurat v3.0 [6]. Then, to denoise the data, we fed the data to SAVER-X, a toolkit implementing an autoencoder combined with a Bayesian method for denoising cross-species data by transfer learning [32]. As a result, the final CBMC dataset contains 8552 cells with 20,501 genes, while the PBMC dataset contains 7947 cells with 17,114 genes.

To train and test the machine learning models, we randomly divided the cells into two disjoint subsets with a 70:30 split for training and testing respectively. Thus, the CBMC training dataset has 5991 cells while the remaining 2561 cells are in the test set. Similarly, the PBMC training and test datasets contain 5567 and 2380 cells respectively. Details of the data are summarized in Table 1.

To incorporate PPI information in the GNN, we selected several PPI features from the STRING database [31] as prior knowledge, including empirically determined interaction, annotated database, automated text mining, combined score, and gene co-occurrence. These features are encoded as floating point numbers.

**Table 1:** Data summary after noise reduction

	CBMC	PBMC
Number of molecular species		
RNA	20,501	17,114
Protein	13	10
Number of cells		
Training set	5991	5567
Testing set	2561	2380
Total	8552	7947

### Analysis of model prediction results

We compared the performance of the proposed PIKE-R2P method with cTP-net [7] and Random Forest [33]. We used the Random Forest available from the Scikit-learn (0.23.1) Python package [34], and the R code of cTP-net. Both PIKE-R2P and Random Forest were trained and tested on the data as summarized in Table 1 with the same input features. However, cTP-net does not provide any training API. Thus, we used the pre-trained cTP-net model with a reduced number of gene expression features $n=12{,}363$, and the performance of cTP-net was evaluated on the testing set only. In addition, cTP-net only predicts 10 proteins in the CBMC dataset, excluding three proteins (CCR7, CCR5, and CD10). Thus, in this section, we also analyzed these 10 proteins only. The performance of the models were evaluated using mean squared error (MSE) and Pearson Correlation Coefficient (PCC) between the ground truth values and the predicted values. For each protein, we picked the best result (i.e. smallest MSE and highest PCC) out of 5 runs. We calculated the means and standard deviations (SDs) for the values of MSE and PCC of the 10 proteins to show the stability of the model.

Table 2 shows the performance of the models on the two datasets. In general, all the models had lower mean MSE and PCC scores on the CBMC dataset than the corresponding scores on the PBMC dataset (except that PIKE-R2P achieved a higher PCC on CBMC than on PBMC). Among the three models, PIKE-R2P got the lowest MSEs on both datasets, the highest PCC on CBMC, and the second highest PCC on PBMC.

**Table 2:** Performance of different models

	CBMC				PBMC			
	MSE	MSE SD	PCC	PCC SD	MSE	MSE SD	PCC	PCC SD
Random forest	0.6608	0.3844	0.5045	0.2675	1.1670	0.9187	0.7459	0.1391
cTP-net	3.1963	1.3963	0.4893	0.4675	3.5971	1.522	**0.8294**	0.1091
PIKE-R2P	**0.2446**	**0.1703**	**0.8640**	**0.0636**	**0.4397**	**0.3360**	0.8144	**0.0999**

The bold numbers represent the best performance among the compared models

**Fig. 1:**
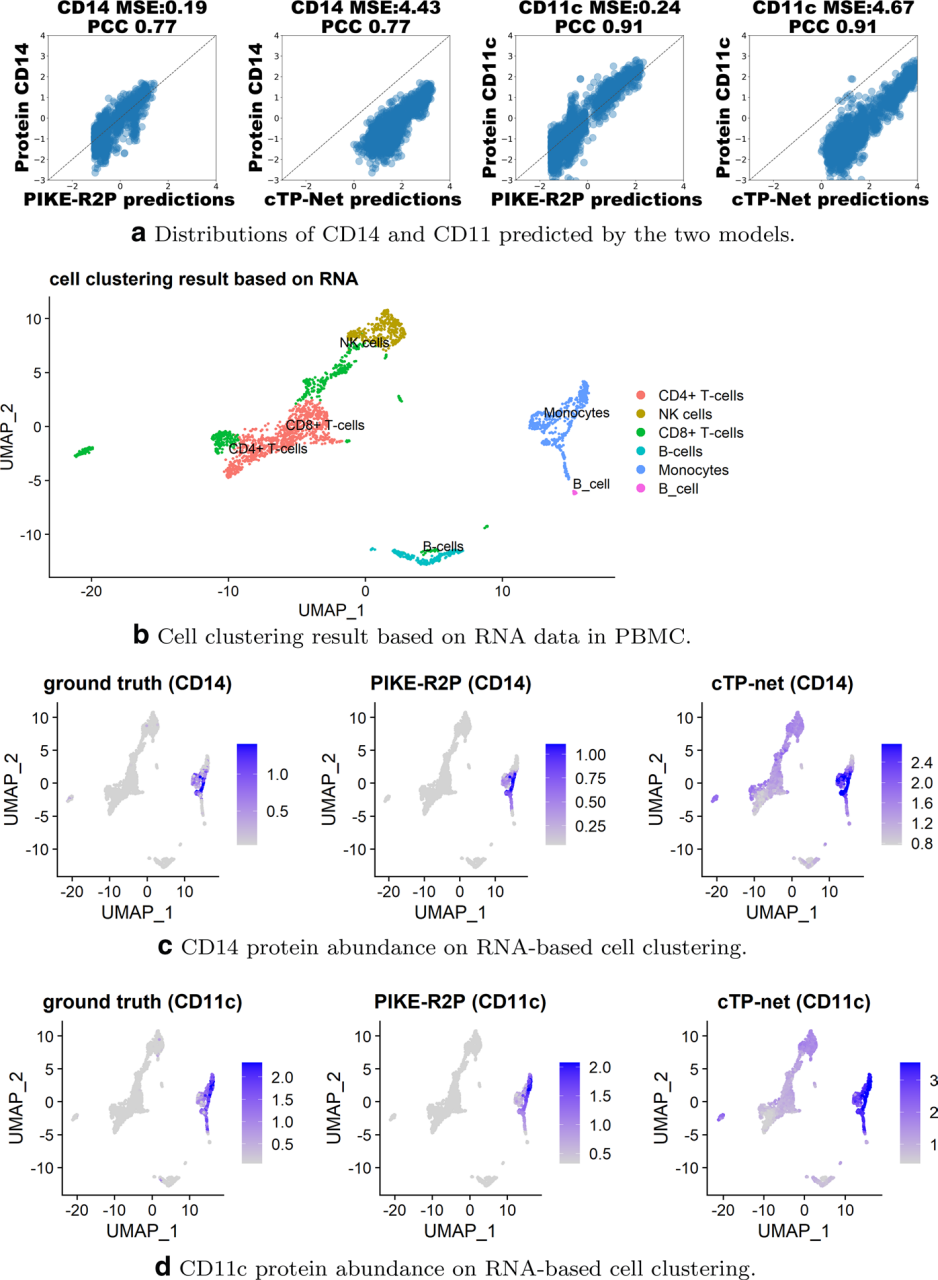
Visualization and comparison of results from cTP-net and PIKE-R2P on PBMC. The visualization results show that the lower the MSE scores, the closer the predicted protein abundances are to the ground truth. In **c** and **d**, from left to right are shown the ground truth, results of PIKE-R2P, and results of cTP-net of protein levels on RNA-based cell clusters in **b**

When the PCC scores are similar, a lower MSE score means the model prediction is closer to ground truth measurement. For example, let us look at the performance of cTP-net and PIKE-R2P on proteins CD14 and CD11c in PBMC. Interestingly, both models agreed that the PCC score of CD14 is 0.77 and that of CD11c is 0.91. However, for CD14, the MSE scores of PIKE-R2P and cTP-net are 0.19 and 4.43 respectively and similarly for CD11c. As shown in Fig. 1a, while the PCC scores are equal between the two models, the predictions of cTP-net deviate from the diagonal, which means the predicted abundance is higher than the ground truth. Using Seurat v3.0 [6], we divided the cells into different cell types based on RNA expression levels as shown in Fig. 1b. Furthermore, Fig. 1c, d show that CD14 and CD11c have high abundance values in Monocytes in the real measurement, which has been successfully captured by PIKE-R2P. However, the predictions by cTP-net have high values for the two proteins in almost all of the cells.

**Fig. 2:**
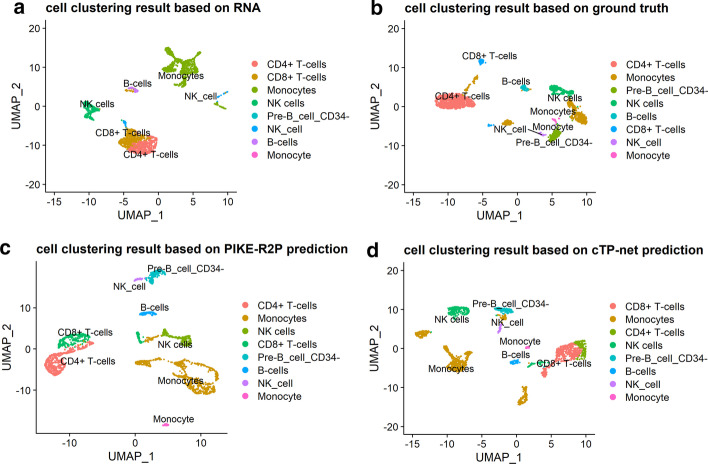
Visualizations of cell clustering results based on different data. The data predicted by PIKE-R2P disperses different cell clusters almost equally well as the ground truth protein data on the CBMC dataset. **a** cell clustering result based on RNA, SC = 0.069; **b** cell clustering result based on ground truth measurement of protein abundance, SC = 0.305; **c** cell clustering result based on PIKE-R2P prediction, SC = 0.309; **d** cell clustering result based on cTP-net prediction, SC = 0.135

To test whether clustering based on the protein data can distinguish cell types more accurately than that based on RNA data, we compared cell clustering results based on the protein abundance values both of ground truth and predicted by PIKE-R2P to RNA-based clustering, and the results are shown in Fig. 2. To cluster the cell types, we used the method of UMAP as implemented in the Seurat v3.0 package. UMAP reduces the dimensionality of data to visualize clustering results [35]. Besides, we calculated the Silhouette Coefficient (SC) scores as a quantitative metric to evaluate the performance of clustering. In Fig. 2a, we find that, when using the RNA data to cluster the cells, CD8$^{+}$ T cells and CD4$^{+}$ T cells are mixed in the same cluster, but when using the ground truth protein data to cluster the cells in Fig. 2b, CD8$^{+}$ T cells and CD4$^{+}$ T cells are in two different groups. Moreover, NK cells, Monocytes, and Pre-B cells in the CBMC dataset are difficult to distinguish with RNA-based clustering as shown in Fig. 2a. By contrasts, in the clustering result based on the ground truth protein data as in Fig. 2b, those three cell types are well separated. Using the protein abundances predicted by PIKE-R2P, the cell types can also be easily distinguished from each other, as shown in Fig. 2c. Using the protein abundances predicted by cTP-net, however, CD8$^{+}$ T cells and CD4$^{+}$ T cells in CBMC are still mixed, as shown in Fig. 2d.

**Fig. 3:**
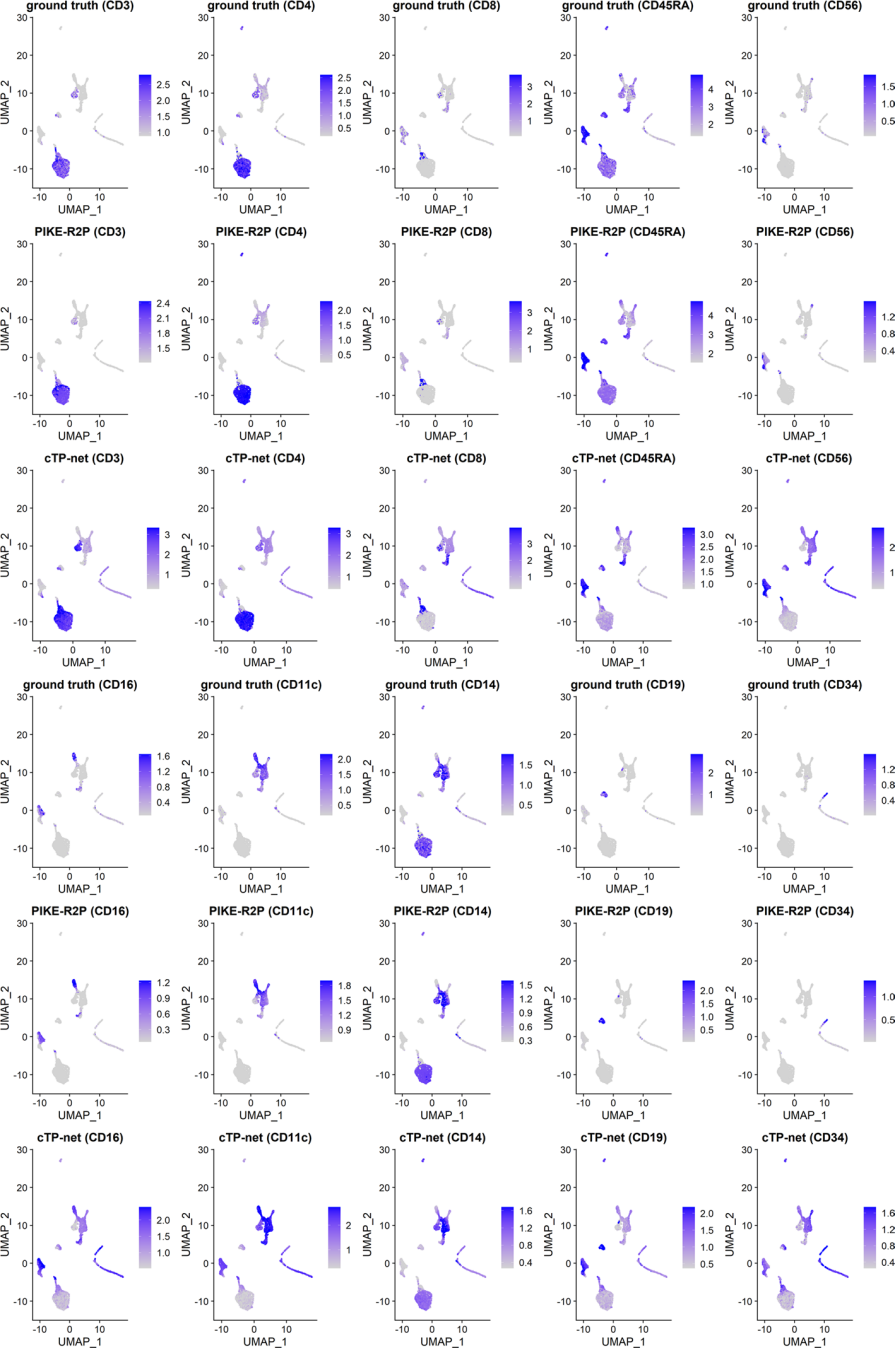
Protein levels on RNA-based cell clustering results on CBMC data. The results predicted by PIKE-R2P are more similar to the ground truth than cTP-net

Protein abundance levels from the ground truth and the predictions of two models are visualized on RNA-based cell clustering in Fig. 3. We find that, for most proteins predicted by PIKE-R2P, the distribution of protein levels across the cell clusters is similar to the ground truth. Each protein is highly expressed in its corresponding cell type annotated based on RNAs. For example, in the ground truth, CD3 is highly expressed in T cells and monocytes, and CD8 is highly expressed in CD8$^{+}$ T cells and NK cells. In this regard, our PIKE-R2P model is able to make predictions similar to the ground truth. However, it is not the case for cTP-net. For instance, cTP-net predicts that CD3 is highly expressed in NK cells and Pre-B cells, and so is CD8 in monocytes. The protein abundances predicted by cTP-net tend to be high on most cell types, which makes it difficult to distinguish the cell types by the predicted protein abundances.

### Module analysis

For noise reduction, we used the pre-trained model of SAVER-X to process the original data. SAVER-X is a self-supervised learning model based on auto-encoder. The pre-trained model of SAVER-X has somehow captured the distributions of RNAs among single cells, and thereby it could filter out some noise that could have made the data not fit the distributions well. Compared with the results without using SAVER-X, we found that the data pre-processing using SAVER-X significantly improved the performance of our model, and made our model converge faster (data not shown).

We further investigated the influence of prior knowledge on the PIKE-R2P model. Our experiment included seven conditions, i.e. no prior knowledge, adding empirically determined interaction, database annotated, automated text mining, combined score, gene co-occurrence, and merging with these five kinds of prior knowledge. To even out the fluctuations of result due to random initialization of the parameter values, we did 5 repeated experiments in each case. Besides, to reduce the effect of overfitting, we ran 450 epochs in each case, and keep the minimum MSE value among the epochs as defined in Eq. 9. For all the experimental results of each group, we calculated the average between the maximum and the minimum values of the scores among the 5 runs and gave the difference between the maximum score and the average in each group of experiments.

The results are shown in Table 3. In general, adding prior knowledge can slightly improve the model performance. For different features, if the prior knowledge reflects biological characteristics, such as combined score, empirically determined interaction, and gene co-occurrence, the model improves more than others. When merging all the 5 types of prior knowledge features, the performance of the model improves the most. However, the scores are very close to each other among the conditions in Table 3. One reason could be that the knowledge information is far less rich than the RNA data, and thus the RNA data are in a dominant position.

**Table 3:** Impact of prior knowledge embedding on model performance of PIKE-R2P

	CBMC		PBMC	
	PCC	MSE	PCC	MSE
No prior knowledge	0.8452 ± 0.0020	0.1960 ± 0.0022	0.8119 ± 0.0049	0.4432 ± 0.0043
Empirically determined interaction	**0.8464** ± 0.0011	0.1958 ± 0.0018	0.8159 ± 0.0038	0.4306 ± 0.0073
Automated text mining	0.8456 ± 0.0011	0.1953 ± 0.0014	0.8165 ± 0.0012	0.4337 ± 0.0055
Database annotated	0.8460 ± 0.0031	0.1957 ± 0.0018	0.8163 ± 0.0030	0.4320 ± 0.0068
Combined score	0.8459 ± 0.0029	0.1952 ± 0.0060	0.8162 ± 0.0020	0.4333 ± 0.0072
Gene co-occurrence	0.8442 ± 0.0012	**0.1944** ± 0.0027	0.8165 ± 0.0019	0.4329 ± 0.0039
Merge 5 features	**0.8462** ± 0.0037	**0.1944** ± 0.0035	**0.8181** ± 0.0013	**0.4303** ± 0.0083

The bold numbers represent the best performance. Note that on the CBMC dataset, for either PCC or MSE, the best and the second best scores are very close to each other, so both results are in bold

**Fig. 4:**
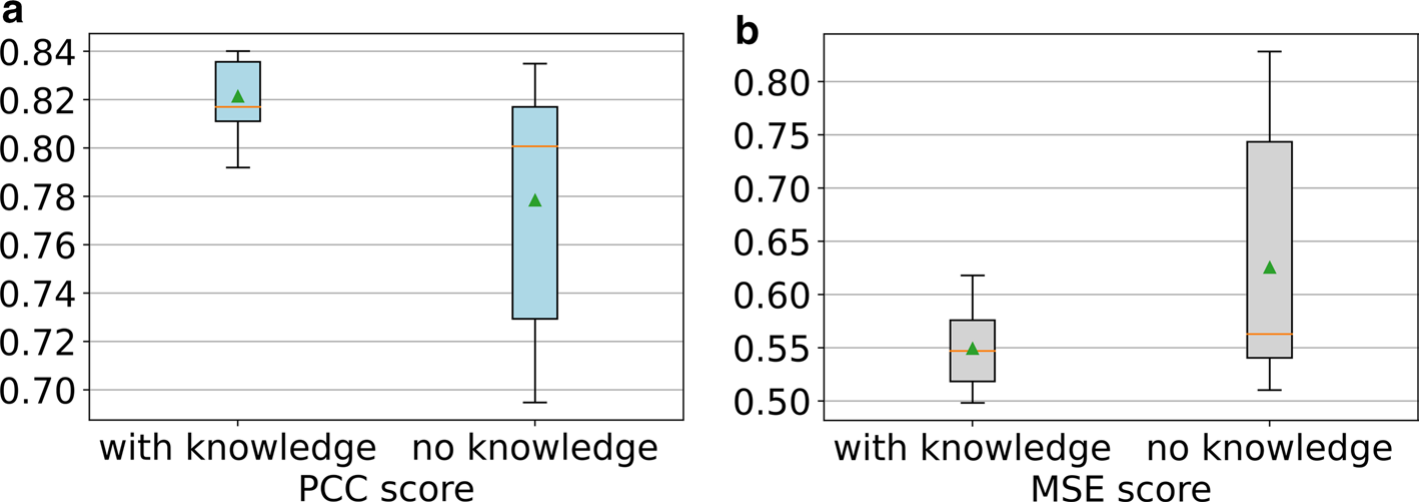
The effect of the knowledge on the performance of PIKE-R2P on the artificial dataset merging CBMC and PBMC. **a** The PCC scores. The median and mean PCC scores are 0.8170 and 0.8214 with knowledge but they are 0.8008 and 0.7784 without knowledge. **b** The MSE scores. The median and mean are 0.5471 and 0.5494 with knowledge but are 0.5631 and 0.6254 without knowledge. In each boxplot, the green triangle marks the position of the average value, and the orange line makes the median value

To further illustrate the power of adding the prior knowledge, we conducted an experiment by merging the two datasets (i.e. CBMC and PBMC) into one artificial dataset, comprising 16,603 types of RNA that overlap between CBMC and PBMC (i.e. the intersection). Then, we added the training sets from CBMC and PBMC together to get 11,558 cells in the merged training set; likewise, we got 4941 cells in the merged test set. We ran PIKE-R2P 15 times for both the condition of using no prior knowledge and the condition of adding prior knowledge with all the 5 features. The box plots in Fig. 4 show that adding prior knowledge can significantly improve the performance of our model on the merged dataset. The results also show that the variances of both PCC and MSE of the model without prior knowledge are larger than the model with knowledge embedding.

## Discussion

In our experiments, Random Forest was more computationally expensive than the neural network-based models (data not shown). This could be due to the sharing of RNA features among different proteins which are reused by neural network models so that some of the model retraining can be avoided, whereas the Random Forest method does the whole feature engineering for every target protein.

We have used the PPI network as prior knowledge. Similarly, several other sources of prior information are available in the literature, including gene ontologies and text mining databases. Each data source could provide additional information while reducing inherent noise in the data. As a future extension, the incorporation of multiple data sources in the model may provide a better prediction framework.

In our work, we predicted proteins using the CITE-seq dataset, where the measurements were performed on blood samples. It has been shown that single-cell gene expression patterns tend to be tissue specific [7, 32]. A transfer learning framework may help train a model from a large known dataset of one tissue while predicting gene expressions in other tissues. A similar approach of transfer learning could also be used to compare different sequencing platforms (e.g. CITE-seq and REAP-seq). In both cases, a model based on graph neural networks incorporating prior knowledge may provide good model performance and biological insights.

## Conclusion

Recently emerging single-cell multi-omics techniques can measure RNA and protein abundances simultaneously in the same cells. Based on such data, machine learning models have been proposed to predict protein abundances based on RNA abundances at the single-cell level. However, their performances can be further improved.

In this paper, we proposed PIKE-R2P, a machine learning method based on graph neural network (GNN) and knowledge embedding. The key idea is that target proteins often share mechanisms of gene expression regulation from transcription to translation. PIKE-R2P captures such relations by embedding the prior knowledge of protein–protein interactions into a GNN. Through information propagation among nodes of the GNN, the model can make better use of information from the RNA-seq data, and thereby improve its prediction performance. Our results on real CITE-seq data demonstrated that PIKE-R2P significantly out-performed existing methods, indicating the value of adding knowledge to neural network models. In the future, more sources of knowledge and more modalities of single-cell data can be integrated through GNN, not only improving prediction performance, but also paving the way for interpretable machine learning in bioinformatics.

## Methods

### Overview

**Fig. 5:**
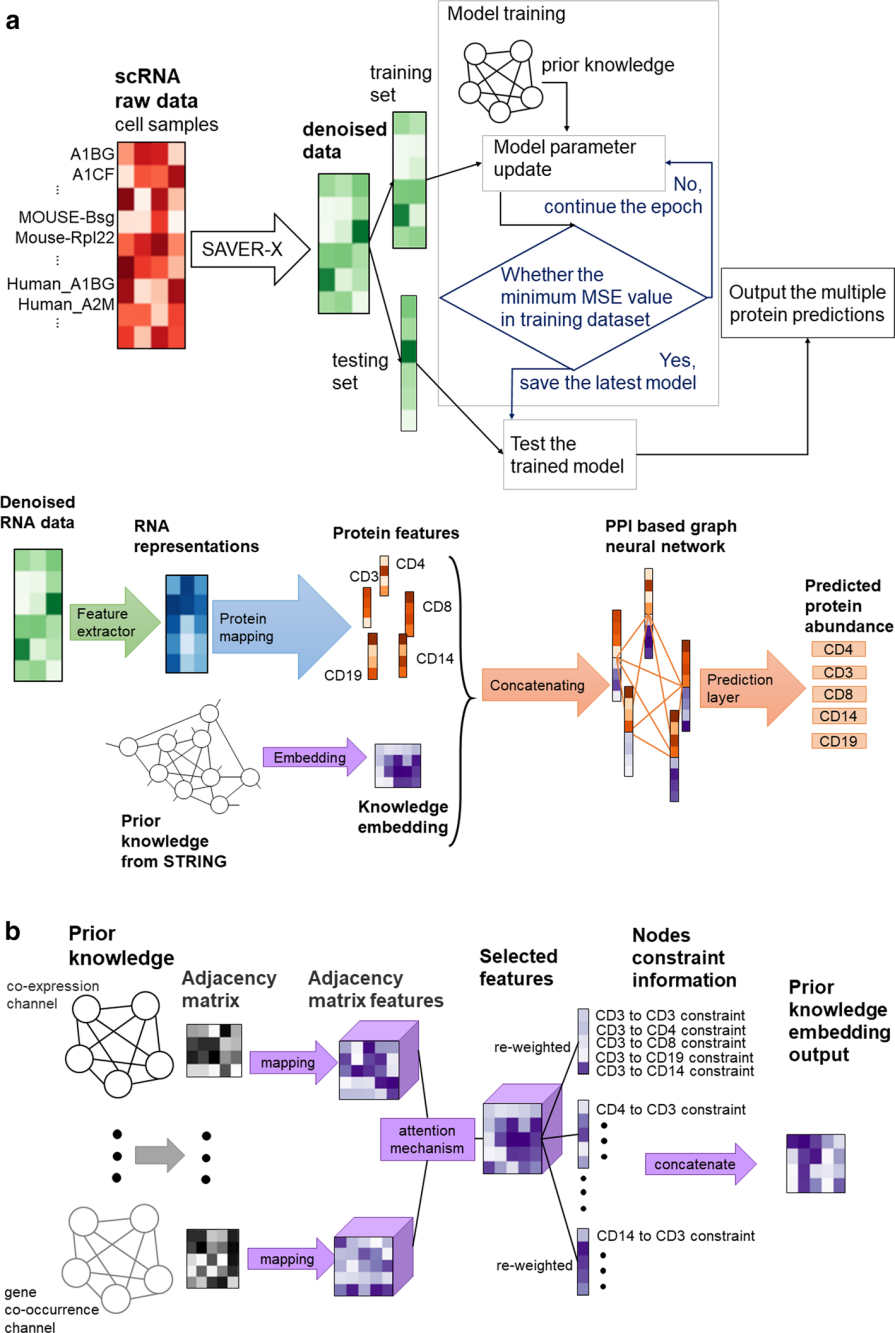
The method of PIKE-R2P. **a** is the whole pipeline and the model structure. The pipeline includes data denoising, model training, and testing. The green matrix represents denoised RNA data; the blue matrix is the high-dimensional representation of the RNA data; the orange vectors are the features of proteins and the orange lines correspond to the edges in the PPI network; the purple vectors are representations of the prior knowledge. **b** The knowledge embedding structure. The prior knowledge of each protein is mapped into a high-dimensional feature matrix. Then the attention mechanism is used to select the features. After that, the weights of protein interaction pairs linked to the same protein are adjusted. Finally, these feature vectors are concatenated together into one matrix as the prior knowledge embedding

The main idea of our method is to integrate the PPI-based information as prior knowledge into a graph neural network, to capture the relationships between proteins and RNAs as well as among proteins, and thereby to improve the accuracy of protein abundance prediction. The whole pipeline is described in Fig. 5a and Algorithm 1. After noise reduction by SAVER-X, we divide the cells into two disjoint datasets, i.e. a training set and a test set. For training, we feed the training set to the model for parameter estimation and save the parameter values that correspond to the minimum MSE loss among all the epochs that have been computed. During the test, the model loads these parameters, and predicts the protein abundances of the cells in the test set directly.

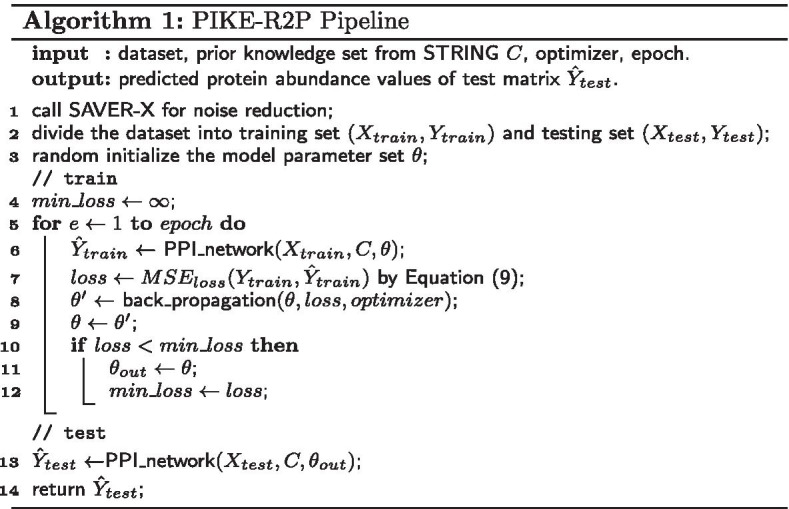



Our model mainly consists of two modules. The first one is adding the PPI-based graph neural network to the dataset, shown as the “PPI-based graph neural network part” in Fig. 5a. These protein–protein interactions provide a way for information transmission between proteins, which means the proteins jointly promote specific biological functions, e.g. by inhibiting or promoting each other [31]. Intuitively, we encode the PPIs with a graph structure, where the nodes are proteins, and edges represent the interactions. Thus, we use the graph neural network to compute the result of information transmission through these interactions between proteins. The other module is the embedding of prior knowledge, such as co-expression and gene co-occurence, etc., which is described in Fig. 5a. Since PPI relationships tend to be conserved across different cell types [31], the PPI in large-scale databases such as STRING can be used for the knowledge embedding.

The whole structure of the model is shown in Fig. 5a. The input is the denoised data from SAVER-X. Then, similar to cTP-net [7], we extract the RNA representation from the input RNA data using a neural network for feature extraction, which includes two fully-connected layers, shown as the blue part in Fig. 5a. After that, to represent the features of *N* proteins in the high-dimensional space independently, we used *N* 1-layer forward networks to map the RNA representation to *N* protein feature vectors, and combined all the feature vectors of the proteins into matrix $V_r\in {\mathbb {R}}^{N\times {d_r}}$, where $d_r$ is the number of dimensions of the protein representations, shown as the orange vectors in Fig. 5a. Besides, the prior knowledge from different sources is embedded into matrix $V_k\in {\mathbb {R}}^{N\times {d_k}}$, where $d_k$ is the number of dimensions of the target vector space of the knowledge embedding, shown as the purple matrices in Fig. 5a. By concatenating the column vectors from the two matrices that correspond to the same protein, the high-dimensional representation of each protein is

1 $$\begin{aligned} v_i=v_{r_i}\oplus {v_{k_i}}, \end{aligned}$$

where $v_i\in {\mathbb {R}}^{1\times {d}}, i=1,2,\dots ,N$, $d=d_r+d_k$ and $\oplus$ is the concatenation operation. Thus, the PPI network has the set of nodes $V=\{v_1,v_2,\dots ,v_N\}$, and $V\in {\mathbb {R}}^{N\times {d}}$. Moreover, the interactions between the proteins are represented as the set of edges $E\subseteq {V\times {V}}$. Therefore, graph $G= (V, E)$ represents the PPI network, as shown in the PPI-based Graph Neural Network part in Fig. 5a. To model the information transmission in the PPI network, we apply algorithms of graph neural network on *G*. After that, to map the *N* representations in *d* dimensions to the abundance values $\hat{Y}\in {\mathbb {R}}^{N\times {1}}$, we reduce the dimensions of the node vectors from *d* to 1 through the predictor which is a 1-layer feed-forward network.

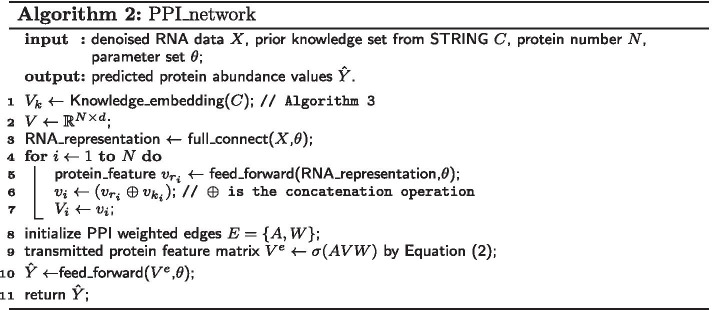



### PPI-based graph neural network

In this paper, we assume that the proteins whose abundances are to be predicted have some relations with each other. Such relations could be due to physical interactions, crosstalk between signaling pathways, shared mechanisms of gene regulation from transcription to translation, or some other functional relationships. For convenience, we consider such relations as “protein–protein interactions” (PPIs) in the general sense, i.e. the PPIs include both direct and indirect interactions. A PPI network is naturally represented as an undirected graph denoted by $G = (V, E)$, where each node in *V* corresponds to a protein and each edge in *E* corresponds to the interaction between two proteins.

To represent the edges in set *E*, we use a weight matrix $W\in {\mathbb {R}}^{d\times {d}}$ to capture the relations among the features of the proteins and we use an adjacency matrix $A\in {\mathbb {R}}^{N\times {N}}$containing edge weights to describe the connectivity among the proteins. The values in both matrices are initialized randomly and will be adjusted when the model is trained, according to the definition of graph neural network in [36]. During the training, the nodes transmit feature information to each other, and the result is:

2 $$\begin{aligned} V^{e}=\sigma (AVW), \end{aligned}$$

where matrix $V^e\in {\mathbb {R}}^{N\times {d}}$ contains the node vectors transformed from the node vectors in *V* through *A*, *W* and the sigmoid function $\sigma (x)=\frac{1}{1+e^{-x}}$, which is applied to each element of matrix *AVW*. After that, we use a Feed-Forward (FF) layer to reduce the dimensions of the node features from $N\times {d}$ to $N\times {1}$, where *N* is the number of proteins. Different from cTP-net [7], which fits the Centered Log-ratio Range of protein abundance [4] by the ReLu function $ReLu(x)=max(0,x)$, we use the PReLu function $PReLu(x)=max(0,x)+0.25\times min(0,x)$ in the last layer to ensure that the model can predict values less than 0. Note that, in the CITE-seq data, the protein abundance values are log-transformed and thus could be negative sometimes. Thus, the output is

3 $$\begin{aligned} \hat{Y}=PReLu(FF(V^{e})). \end{aligned}$$

### Prior knowledge

In the previous section we mainly built a PPI network from a specific dataset, but there is additional prior knowledge about PPI from other datasets. The STRING database collects information on PPI from different anngles such as co-expression and gene co-occurrence, etc. Therefore, we use this superset of PPI information to improve the model performance. To represent these features, we embed this prior knowledge into $d_k$ dimensions, which adds constraints to the protein predictions in the graph neural network. The structure is shown in Fig. 5b and the algorithm is described in Algorithm 3.

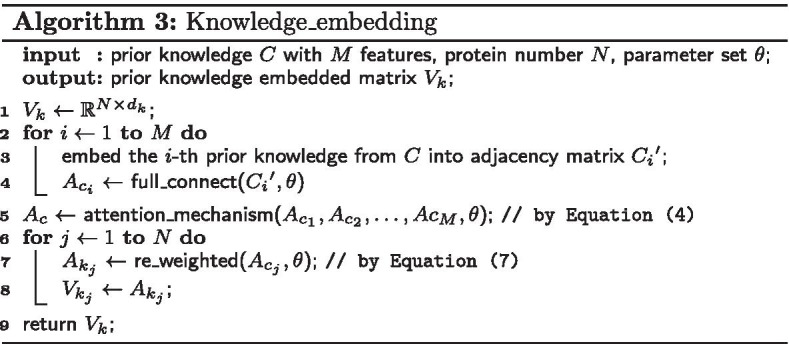



We use *M* independent features $C=\{C_1,C_2,\dots , C_M\}$ of the PPIs in the STRING database [31]. Each feature $C_i$ is represented by a graph with *N* protein nodes and $N\times {N}$ edges represented by the interaction scores, where *N* is the number of proteins. We transform every $C_i$ into an $N \times N$ adjacency matrix ${C_i}'\in {\mathbb {R}}^{N\times {N}\times {1}}$. When a protein is missing in the prior knowledge database, which means the connections of the protein with others are absent. We set the weights of the connections to 0. In order to obtain the high-dimensional features of each adjacent matrix, each column vector in matrix ${C_i}'$ is encoded by *N* 1-layer fully-connected networks with $d_c$ dimensions and the result is $A_{c_i}\in {\mathbb {R}}^{N\times {N}\times {d_c}}$. Then, through the attention mechanism defined in [37], the importance scores of the features are merged into matrix $A_c\in {\mathbb {R}}^{N\times {N}\times {d_c}}$,

4 $$\begin{aligned} A_c &= elu\left( \frac{1}{M}\sum _{i=1}^{M}(a_{c_i}W_{a_i}A_{c_i})\right) , \end{aligned}$$

5 $$\begin{aligned} a_{c_i}&= \frac{\mathrm{exp}{(elu(A_{c_i}))}}{\sum _{e=1}^{N}\mathrm{exp}{(elu(A_{c_e}))}}, \end{aligned}$$

where $a_{c_i}$ is the normalized attention coefficient, $W_{a_i}$ is the weighted matrix for the *i*-th coefficient, and $elu(x)=max(0,x)+min(0,\mathrm{exp}(x)-1)$.

To combine the prior knowledge with each protein node to constrain the information transmission, we divide $A_c$ into *N* submatrices $A_{c_j}\in {\mathbb {R}}^{N\times {d_c}}$, where $0<j\le {N}$, and each submatrix corresponds to one of the *N* proteins. To reflect different degrees of importance of the protein pairs, we need to re-weight all the relationships. In the following, $A_{k_j}\in {\mathbb {R}}^{N\times {d_c}}$ represents the re-weighted relationships:

6 $$\begin{aligned} a_{k_j}&= \frac{\mathrm{exp}{(elu(A_{k_j}))}}{\sum _{e=1}^{N}\mathrm{exp}{(elu(A_{k_e}))}}, \end{aligned}$$

7 $$\begin{aligned} A_{k_j} & = elu(a_{k_j}W_{k_j}A_{c_j}), \end{aligned}$$

where $a_{k_j}$ is the normalized attention coefficient for the different constrained features. Because a pair of proteins may be influenced by multiple intermediate proteins, we concatenate all the prior knowledge of protein interactions for each node into a feature vector, as follows:

8 $$\begin{aligned} V_k=A_{k_1}\oplus {A_{k_2}}\oplus \dots \oplus {A_{k_N}}, \end{aligned}$$

where $V_k\in {\mathbb {R}}^{N\times {d_k}}, d_k=N\times {d_c}$, and $\oplus$ is the concatenation operation.

### Model training

Before training, we set the parameters for the model. In the fully connected layers, the hidden sizes are 1024 and 128 for the numbers of output neurons of the two hidden layers for the RNA representation and 32 hidden neurons in the connected layer for the prior knowledge embedding. In the feed-forward network, we set $d_r$ to 64, $d_c$ to 32 and $d_k$ to $d_c \times N$. The number of nodes *N* in our graph neural network depends on the dataset, i.e., $N=10$ for PBMC and $N=13$ for CBMC. Thus, $d_k=320$, $d=d_r+d_k=384$ for PBMC, and $d_k=416$, $d=d_r+d_k=480$ for CBMC.

For the training, we set the number of epochs to 350 and batch size to 32. For the optimization of loss function based on mean squared error (MSE), we first set the global $MSE_{loss}'$ to an infinite value. In each epoch, if the current $MSE_{loss}$ is smaller than the global $MSE_{loss}'$, we update $MSE_{loss}'$ to $MSE_{loss}$, and save the model parameters of this epoch. We assume that all proteins have equal weights in the MSE loss:

9 $$\begin{aligned} MSE_{loss}(Y,\hat{Y})=\sum _{i=1}^{N}{(\hat{y}_i-y_i)}^2, \end{aligned}$$

where *Y* contains the ground truth measurements and $\hat{Y}$ is the set of the predicted protein abundances. The initial learning rate is set to $10^{-6}$. The model parameters are estimated based on the minimization of MSE loss and the Adam optimizer by back propagation.

## Data Availability

The raw data is at https://www.ncbi.nlm.nih.gov/geo/query/acc.cgi?acc=GSE100866. The code is available at https://github.com/JieZheng-ShanghaiTech/PIKE-R2P.

## Abbreviations

PIKE-R2P: Protein–protein interaction network-based knowledge embedding with graph neural network for single-cell RNA to protein prediction
PPI: Protein–protein interaction
CBMC: Cord blood mononuclear cells
PBMC: Peripheral blood mononuclear cells
PCC: Pearson correlation coefficient
MSE: Mean squared error
SD: Standard deviation
GNN: Graph neural network
